# A Full Lifecycle Bioenergetic Model for Bluefin Tuna

**DOI:** 10.1371/journal.pone.0021903

**Published:** 2011-07-11

**Authors:** Marko Jusup, Tin Klanjscek, Hiroyuki Matsuda, S. A. L. M. Kooijman

**Affiliations:** 1 Faculty of Environment and Information Sciences, Yokohama National University, Yokohama, Japan; 2 Department for Marine and Environmental Research, Rudjer Boskovic Institute, Zagreb, Croatia; 3 Department of Theoretical Biology, Vrije Universiteit, de Boelelaan, Amsterdam, The Netherlands; Vrije Universiteit, Netherlands

## Abstract

We formulated a full lifecycle bioenergetic model for bluefin tuna relying on the principles of Dynamic Energy Budget theory. Traditional bioenergetic models in fish research deduce energy input and utilization from observed growth and reproduction. In contrast, our model predicts growth and reproduction from food availability and temperature in the environment. We calibrated the model to emulate physiological characteristics of Pacific bluefin tuna (*Thunnus orientalis*, hereafter PBT), a species which has received considerable scientific attention due to its high economic value. Computer simulations suggest that (i) the main cause of different growth rates between cultivated and wild PBT is the difference in average body temperature of approximately 6.5°C, (ii) a well-fed PBT individual can spawn an average number of 9 batches per spawning season, (iii) food abundance experienced by wild PBT is rather constant and sufficiently high to provide energy for yearly reproductive cycle, (iv) energy in reserve is exceptionally small, causing the weight-length relationship of cultivated and wild PBT to be practically indistinguishable and suggesting that these fish are poorly equipped to deal with starvation, (v) accelerated growth rate of PBT larvae is connected to morphological changes prior to metamorphosis, while (vi) deceleration of growth rate in the early juvenile stage is related to efficiency of internal heat production. Based on these results, we discuss a number of physiological and ecological traits of PBT, including the reasons for high Feed Conversion Ratio recorded in bluefin tuna aquaculture.

## Introduction

Bluefin tuna (family *Scombridae*, tribe *Thunnini*, genus *Thunnus*) has received considerable scientific attention on individual and population levels [1]–[3]. At both levels, nonetheless, poorly understood issues are still identifiable. At individual level, feeding and reproduction are being studied to create ecologically and economically viable aquaculture as an alternative to traditional fishing [4], [5]. At population level, effectiveness of stock assessment and management methods is being debated to prevent overexploitation [6], [7]. Existing knowledge on tunas partly addresses the issues involved, but lacks an integrative approach that would link observations at individual and population levels. A possible reason for difficulties in the integration of knowledge may be the interdisciplinary origin of available information such as evolutionary or comparative physiology [8]–[10] and experimental bioenergetics [11]–[15]. We believe, however, that recent developments in Dynamic Energy Budget (DEB) theory provide a generalized, individual-based, bioenergetic framework [16] suitable for linking levels of metabolic organization [17]. In DEB theory, macrochemical transformations within organisms are reflected in mass and energy balances that enable useful insights into physiological and ecological traits of individuals and populations likewise. In addition, DEB theory accounts for all life stages including the embryonic development [18], is applicable to all species including fishes [19]–[24], and can relate ecotoxicology to organismal bioenergetics [25], [26]. Herein, we exploit the strengths of DEB framework in order to (i) encapsulate all life stages of bluefin tuna in a single bioenergetic model that allows for the comparison of energy allocation between different stages, (iii) use the model to examine various physiological and ecological traits of bluefin tuna, (iv) identify key research questions that may significantly improve our understanding of bluefin tuna physiology and ecology, plus (iv) lay foundations for future modeling efforts that will bridge the gap between the individual- and population-based models.

In the next section, we present a detailed model description, together with parameter estimation procedure and a list of data sources used to estimate the parameters. We proceed with an overview of results, demonstrating the extent to which the model was successful in reproducing the existing data and illustrating various predictive capabilities of the model. Particular attention is given to comparison of PBT growth in the aquaculture and in the wild. An extensive discussion reviews physiological and ecological traits of PBT revealed by the model. Finally, a brief conclusion offers a fresh look on PBT emanating from this research.

## Methods

### Model formulation

Relying on the principles of DEB theory [16], and following the work of Pecquerie [19], [20], we conceptually divide the fish into two compartments distinguished by their dynamics: reserve and structure. One can intuitively think of reserve as the mass composed of all compounds that can be metabolized to fuel metabolic processes. Reserve responds directly to changes in food availability and does not require maintenance. In contrast, structural mass is necessary to conduct basic life functions such as finding and handling prey. The increase of structural mass depends on the current amounts of reserve and structure, while the turnover of previously built structure imposes continuous maintenance costs. Using this conceptual division, we introduce two model state variables: *E* – the amount of energy in reserve and *V* – volume of structural mass. Structural volume can also be characterized by structural length (*L*), such that 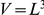. On top of separating the fish into two compartments, the model explicitly accounts for fish maturation. We, therefore, consider the third state variable denoted by 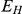 and called the level of maturity (Table 1; see also Appendix A). The formal status of maturity in DEB theory is information, because energy or mass invested into maturation is dissipated in form of heat or metabolites. We avoid the problem of assigning units to information by quantifying maturity in terms of the accumulated invested amount of reserve, which can be tracked in units of energy or mass (J or C-mol).

**Table 1:** List of symbols I: state and auxiliary variables.

Symbol	Definition	Unit
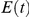	Amount of energy in reserve tissue	J
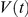	Volume of structural tissue	cm^3^
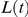	Structural volumetric length	cm
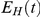	Level of maturity	J
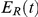	Status of the reproductive buffer	J
*E*_0_	Initial energy reserve of an egg	J

Literature defines four developmental stages of PBT [27]–[29]: embryonic, larval, juvenile and adult. We model stage transitions by increasing the level of maturity 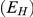 from zero to a maximum value and defining threshold levels at which transitions take place. An embryo becomes a larva at 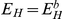, metamorphoses into a juvenile at 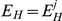, and matures into an adult at 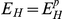. In addition, young juveniles are said to be in the early juvenile phase until the threshold maturity level 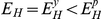. The standard DEB model identifies stage transitions with fundamental changes in the energy budget, i.e. onset of assimilation when an embryo turns into a juvenile and imminent investment into reproduction when a juvenile turns into an adult [16]. In the case of bluefin tuna, growth acceleration in the larval stage and growth deceleration in the early juvenile phase suggest additional fundamental changes in energy allocation, which justify the introduction of non-standard transition thresholds. The level of maturity does not increase in the adult stage, because adults are considered to allocate energy to reproduction. Reserve allocated to reproduction is accumulating in a reproductive buffer in which egg production occurs according to species-specific buffer handling rules [16], [19], [20]. Status of the reproductive buffer is quantified by an auxiliary variable 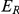 (Table 1). Before entering the adult stage there are no reproductive events and 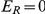.

**Figure 1:**
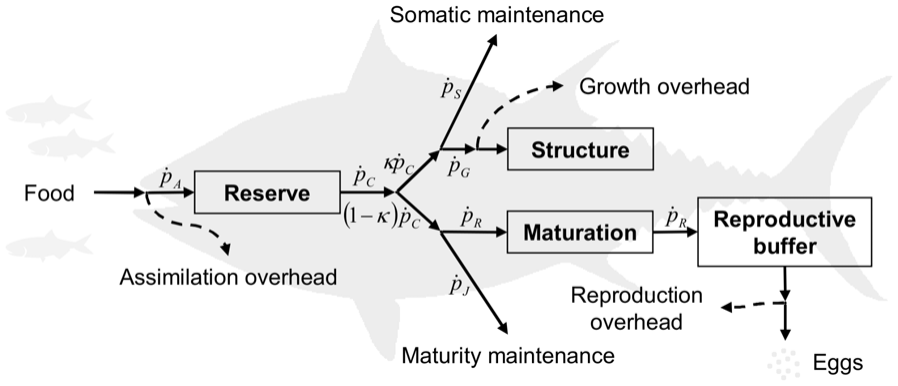
Schematic representation of a dynamic energy budget of Pacific bluefin tuna. Energy fluxes are defined in Table 2. See text for details.

Dynamics of all three state variables and the status of the reproductive buffer are determined by energy fluxes of an individual fish (Figure 1). Detailed specification of all energy fluxes (Table 2) leads to a concisely defined DEB model for PBT (Table 3), which includes several extensions to the standard DEB model (Table 4), to account for growth acceleration in the larval stage, growth deceleration in the early juvenile phase, changes in body shape, and effects of temperature on metabolic rates.

**Table 2:** Dynamic energy budget of Pacific bluefin tuna I: energy fluxes.

Metabolic process	Eq.	Energy flux
Assimilation	F1	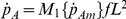
Utilization	F2	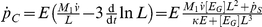
Somatic maintenance	F3	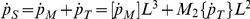
Growth	F4	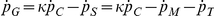
Maturity maintenance	F5	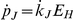
Maturation and reproduction	F6	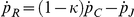

**Table 3:** Dynamic energy budget of Pacific bluefin tuna II: dynamics of state variables and the reproduction rate.

State variable	Eq.	Dynamic equation
Energy in reserve	D1	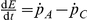
Structural length	D2	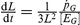
Maturity level	D3	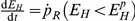
Reproductive buffer^a^	D4	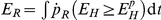

a In equation D4 the integration is being performed from the last reproductive season to the present moment.

**Table 4:** Dynamic energy budget of Pacific bluefin tuna III: auxiliary functions.

Auxiliary function	Eq.	Functional form
Shape correction function	A1	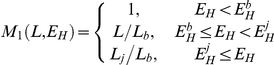
Efficiency of internal heat production	A2	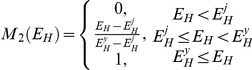
Arrhenius equation	A3	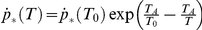
Shape factor	A4	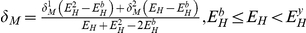

One of the key concepts in DEB theory is homeostasis [16]. Homeostatic mechanisms, in general, tend to maintain stability of internal conditions of an organism even as environmental conditions change. This is primarily reflected in relatively constant chemical composition of the body; fish are no exception [30]. Consequently, the chemical composition of reserve and structure is constant, but their ratio can change, thus affecting the chemical composition of the whole body. If environmental conditions do not vary much, the composition of the whole fish is also constant. Note that this implies the existence of a steady-state in which the ratio of energy in reserve to structural volume does not change along the growth curve (i.e. 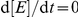, where 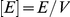).

Ingestion flux is the energy acquired by feeding per unit of time. Closely related to ingestion is the assimilation flux which represents the amount of energy fixed into the reserve per unit of time. It equals the ingestion flux minus the assimilation overhead originating partly from inefficiencies of the digestive system. Efficiency of the digestive system is commonly expressed in terms of Apparent Digestibility Coefficient (ADC). The value of ADC is deduced by comparison of particular compound (e.g. protein) in the diet with that recovered from the feces [31]. ADC, therefore, effectively measures the loss of energy in form of fecal waste. From the perspective of DEB theory, there are additional losses before energy is assimilated by the fish. These losses may include metabolic costs of food acquisition and conversion of food into the energy reserve. When all losses are independent of the feeding rate, assimilation flux remains proportional to ingestion flux. If we further assume that every exchange (including feeding) between the fish and the environment occurs over surfaces which separate the two (skin, feeding apparatus, gills, gut wall etc.), assimilation flux can be represented by equation (F1; Table 2). Holling type II functional response *f* converts food availability to ingestion, or equivalently, assimilation flux. It is a dimensionless quantity that can be written as
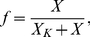
(1)where *X* is food density in the environment and *X_K_* the half-saturation constant. Hyperbolic functional form in (**1**) follows from considerations that the fish needs some time to find and handle a prey before ingesting it. If 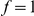, the steady-state of energy reserve density is maximal and denoted by 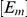.

We assume that energy assimilated into the reserve is utilized for various metabolic processes at a rate determined by the utilization flux (also called the mobilization flux or catabolic power). The utilization of reserve follows from the homeostasis assumption [32], which yields equation (F2; Table 2) for the corresponding energy flux (see also Appendix B). Mobilized reserve is divided according to the κ-rule: a fixed fraction is allocated to the soma, the rest to development and reproduction. In the standard DEB model, metabolic processes and corresponding energy fluxes are:

1. Somatic maintenance, 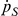 – energy flux to basic metabolic processes that keep the fish alive. We differentiate between structural volume-related maintenance costs 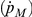 and structural surface-area-related maintenance 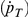. Thus, 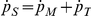.
2. Growth, 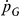 – increase of structure. Growth stands for the change in size, but not complexity (see maturation below). It includes the costs of converting energy reserve into structure 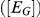, because the chemical composition of two compartments is different.
3. Maturity maintenance, 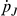 – maintenance of complexity of structure (see maturation below).
4. Maturation, 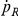 – increase of complexity of structure as a preparation for the adult stage (involving extensive gene regulation switching, cell and tissue differentiation, etc.). Investment into the increase of complexity is not included in the growth flux 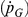.
5. Reproduction, 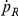 – conversion of mother's energy reserve into the energy reserve of an egg. It is important to note that reproduction flux is a continuation of maturation flux (hence the same notation). We interpret this by assuming that complexity of the organism increases up to a point called puberty, after which energy used for maturation is redirected to production of eggs. This simple assumption effectively explains why reproduction does not cause growth retardation, although it is an energetically intensive process.

Before specifying all fluxes in more detail, we note that the utilization flux can be partitioned as
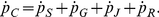
(2)Using a function 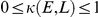, without loss of generality it is possible to write
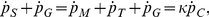
(3)where *κ* is the fraction of utilized energy allocated to somatic maintenance and growth. The rest is allocated to maturity maintenance and maturation or reproduction, i.e.
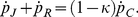
(4)In the standard DEB model the allocation fraction *κ* is constant [16], [32]. We adopt the same approach in development of DEB model for PBT.

Somatic maintenance costs include three major contributions:

1. Structure protein turnover and maintenance of concentration gradients of metabolites across membranes, both proportional to structural volume [16].
2. Energy costs of movement assumed to be proportional to structural volume. Principles of caudal fin propulsion [33] leave the question of exact scaling law open, but do suggest that proportionality to 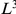 might be a reasonable approximation.
3. Internal heat production and osmotic regulation, both assumed to be proportional to structural surface-area.

The last assumption merits further discussion, especially in relation to internal heat production. Tunas are known for regional endothermy, i.e. a capability to maintain elevated temperature of several tissues in comparison to the surrounding seawater [34]. This property received considerable research interest [35], ultimately leading to thorough investigation of PBT heat budget by means of lumped system thermal analysis [36]–[39]. Results indicate that energetic costs related to regional endothermy scale approximately with the square of structural length, as suggested by DEB theory. Accordingly, we use equation (F3; Table 2) to include somatic maintenance costs into the model.

Full specification of utilization and somatic maintenance fluxes allows us to use equation (3) and express the flux of energy directed to growth by (F4; Table 2).

Two remaining fluxes to be specified are related to fish maturation and reproduction. Investment of energy into maturation increases the complexity of structure as a preparation for the adult stage. At the same time, costs of maintaining newly acquired features increase as well, resulting in equation (F5; Table 2) for maturity maintenance flux. Using relation (4) in combination with (F5; Table 2), we obtain the expression (F6; Table 2) for maturation flux. After the onset of reproduction, complexity does not change since fish grow only in size. Accordingly, maturity maintenance flux becomes constant in the adult stage.

Dynamics of state variables and expression for the reproduction rate (Table 3) can be inferred from Figure 1, Table 2 and the above discussion. The energy in reserve, for example, is determined by the difference of assimilation and utilization fluxes (D1; Table 3). Growth flux regulates the increase of structure (D2; Table 3), while maturation and reproduction fluxes control the level of maturity and the reproduction rate (D3 and D4; Table 3). Calculation of initial energy reserve of an egg, however, is not straightforward and follows from the maternal effect requirement; a method to calculate initial energy reserve of an egg is thoroughly described in literature [16], [18]. We use a numerical implementation of this method, available freely in the DEBtool software package [40].

Since our model specifies the complete life cycle of PBT, it must also account for extraordinary acceleration of growth rate during the larval stage [27]. A similar problem is treated in the case of anchovy (*Engraulis encrasicolus*) in the Bay of Biscay [19], where a shape correction function that depends on maturity level is used to modify the relationship between length and surface area of the fish, thereby increasing the maximum surface-area-specific assimilation rate from birth to metamorphosis. Further work on the same anchovy population [20] uses structural length as domain for the shape correction function, because the level of maturity cannot be linked to organismal size except in one special case (when 
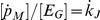
). Changes in shape not only affect assimilation, but also the energy conductance, which occurs in the expression for utilization flux. The dimension of energy conductance is length per time, and this length is in fact the ratio of volume to surface-area; such a ratio is proportional to length only for isomorphs. The consequence is that growth is not only accelerated by a larger intake, but also by a larger mobilization from reserve in comparison to an isomorph. Under this approach, values of surface-area-specific assimilation rate and energy conductance after the metamorphosis depend on the feeding history during the larval stage, which may partly explain natural variability in parameters between individuals of the same species [41]. Actual functional form describing the effects of change in shape used in the present model (A1; Table 4) is not inferred from allometric considerations, but from the observation that growth in the larval stage is approximately exponential.

In the larval stage and early juvenile phase, PBT are unable to raise the body temperature above the surrounding seawater temperature. However, the difference between two temperatures becomes measurable in several tissues (e.g. red muscle, white muscle and peritoneal cavity) as the fish reach 20 cm FL [39]. We model this by gradually introducing the effect of internal heating on the energy budget. First we assume that efficiency of internal heat production remains zero until the beginning of the early juvenile phase at maturity level 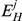. Then the efficiency increases with the level of maturity and reaches a maximum value at critical maturity 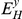, which marks the end of the early juvenile phase. Functional form describing the efficiency of internal heat production is given by equation (A2; Table 4). The function increases linearly from 0 to 1; although no a priori reasons exist to discard the non-linear effect, gradual decrease of growth rate suggests that its role is practically negligible. Inserting function (A2; Table 4) into the equation (F3; Table 2), gives the expression for energetic costs of internal heating.

In our efforts to explain how environmental conditions affect the life histories of PBT, we must also consider the role of temperature. Arrhenius relationship (A3; Table 4), despite its limitations [16], [42], seems to accurately describe the effect of body temperature on metabolic rates within the temperature tolerance range of an individual. In the case of PBT, an additional difficulty in describing the role of temperature arises from partial endothermy. More precisely, due to elevated temperature of various tissues in comparison to the surrounding seawater, we cannot rely on simple measurement of water temperature to be representative of the metabolic rates of an individual PBT. Overcoming this difficulty is made possible by experimental results [37], which indicate that the difference between body temperature 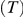 and ambient temperature 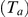 is approximately proportional to the cubic root of body weight; link between the model state variables and body weight is explained in the next subsection. Since various tissues of PBT exhibit various temperatures at the same time, we use peritoneal cavity as a proxy for body temperature. Temperature of peritoneal cavity is lower and exhibits less variance than temperatures of red and white muscles [39].

### Link between DEB state variables and quantities in fisheries science

State variables of DEB-based PBT model can be related to quantities traditionally used in fisheries science: body weight, condition index and batch fecundity. Weight, for example, has contributions from structure, reserve and the reproductive buffer (in adults):
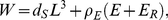
(5)where 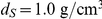 is density of structure and 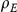 is weight-energy coupler that combines information on molecular weight of generalized reserve compound and its chemical potential (see [16] for more details). By assuming that the amount of structure (and maturity) is negligible at the very beginning of egg development and inserting the initial energy reserve (*E*_0_) into the equation (**5**) it is possible to estimate the weight of a single fish egg, 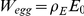. Using data on egg density and presupposing a spherical shape, we also calculate the egg diameter.

Equation (**5**) is particularly useful for constructing the weight-length relationship predicted by the model. However, to make comparisons with the existing data, first it is necessary to relate the structural length of fish to its physical length. In the case of an isomorphic organism, the ratio of any two length measurements is constant throughout the entire life. Therefore, the ratio of structural length to a well-chosen physical length can safely be assumed constant for an isomorph. In the case of PBT, however, relating the physical length to structural length is somewhat problematic, because PBT undergoes a striking transformation during the larval stage. Although young juveniles resemble adult fish after the metamorphosis, data suggest that an isomorphic body shape may not be reached during the first three to four months of development [29]. As a consequence, the shape factor is not constant. To account for the change in shape, we assume that the shape factor increases from an initial value 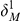, representative of a recently hatched larvae, to the final value 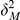, which is representative of an adult individual (A4; Table 4). The relationship describing the change in shape is nonlinear, because the most striking transformation occurs in the early phases of development. The final shape is reached only asymptotically. Parameter 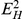 appearing in equation (A4; Table 4) represents the level of maturity at which the shape factor is an arithmetic mean of 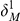 and 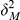. It is accordingly named the half-saturation maturity. We were forced by the available data to relate the structural length to total length (TL) in the larval stage and early juvenile phase, while we worked with fork length (FL) in the later stages. The difference between total and fork length is marginal [29].

The so-called Fulton's condition index [43] is calculated as the ratio of weight to the cube of fish length and sometimes used as a proxy for metabolic reserves. In the case of anchovy in the Bay of Biscay, the condition index increases with fish size [19], while the mean condition index of female PBT from the south-western part of North Pacific drops seasonally between late May and early June [44]. These facts can be accounted for by acknowledging that (i) energy allocated to reproduction throughout the year (i.e. energy in the reproductive buffer, *E_R_*) is stored until the relatively short reproductive season begins, (ii) all stored energy contributes to body weight and (iii) the ratio of energy in the reproductive buffer to structural volume increases with fish size. If we define a condition index by 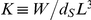, then from equation (**5**) we obtain
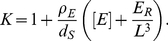
(6)The last expression is, in fact, an increasing function of fish size at constant food level and exhibits seasonal drops that correspond to the reproductive season.

Status of the reproductive buffer, defined by equation (D4; Table 3), can be connected to data on batch fecundity by assuming that energy stored in the buffer throughout the year is rather equally divided among an average number of batches spawned during the reproductive season. In particular, if we denote the number of spawned batches by *N* and batch fecundity by *F*, the relation 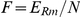 holds, where the index *m* denotes the status of the reproductive buffer just prior to spawning.

### Parameter estimation and data sources

To estimate parameters and calibrate the model, we had to rely on indirect and data intensive methods [40], [45]. In the case of PBT, we used time to hatching as a function of temperature, growth curves of cultivated PBT in all life stages, weight-length relationship for juvenile PBT, batch fecundity vs. fork length and some univariate data (i.e. information on freshly spawned egg size, length at hatching, age at maturity and maximum length). To find values of basic DEB (Table 5) and other (Table 6) parameters that produce the best fit of model predictions to data, we relied on the Nelder-Mead numerical optimization method available in the DEBtool software package [40].

**Table 5:** List of symbols II: basic DEB parameters.

Symbol	Definition	Value and unit
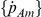	Maximum surface-area-specific assimilation rate	224.0 J·cm^−2^·d^−1^
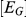	Volume-specific cost of structure	8828 J·cm^−3^
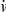	Energy conductance	0.330 cm·d^−1^
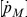	Volume-specific somatic maintenance rate	17.9 J·cm^−3^·d^−1^
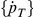	Surface-area-specific somatic maintenance rate	2282 J·cm^−2^·d^−1^
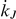	Maturity maintenance rate coefficient	6.303·10^−2^ d^−1^
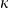	Fraction of mobilized reserve allocated to soma	0.7807
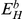	Maturity at birth	0.7637 J
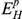	Maturity at puberty	2.548·10^7^ J

**Table 6:** List of symbols III: other parameters.

Symbol	Definition	Value and unit
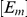	Maximum reserve energy density	680 J·cm^−3^
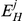	Maturity at the end of the larval stage	6.902·10^3^ J
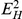	Half-saturation maturity	5.402·10^5^ J
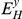	Maturity at the end of the early juvenile phase	9.695·10^5^ J
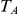	Arrhenius temperature	5300 K
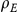	Weight-energy coupler	1.288·10^−4^ g·J^−1^
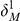	Shape factor in the larval stage	0.2249
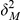	Shape factor in the adult stage	0.2704
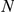	Average number of spawned batches during the reproductive season	9

The Nelder-Mead method requires a user to provide initial values for all parameters subject to estimation, but these values do not affect the final output. The reason is that if the set of chosen initial values falls into the basin of attraction of the Nelder-Mead procedure, the algorithm will always converge to the same final values. In order to ensure the physiological plausibility of estimated parameters, initial values were extracted from available data where possible (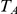, 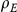, 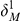, 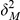 and 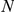), while other initial values were set based on the primary scaling relationships prescribed by the standard DEB model [16].

Embryonic development of PBT and effects of water temperature on hatching were thoroughly discussed in literature [28]. In particular, incubation time was measured in the temperature range between 19.9 and 31.5°C. We used this data, together with information on length at hatching, to obtain the value of maturity at birth 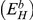 and Arrhenius temperature 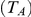; the latter parameter determines the effect of temperature on metabolic rates.

Growth of PBT larvae [4], <30 Days After Hatching (DAH), provided direct access to maturity at the end of the larval stage 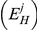. Recorded seawater temperatures, in which PBT larvae were reared, ranged from 24.5 to 27.5°C.

Growth from 30 to 120 DAH [29] was used to estimate the maturity at the end of the early juvenile stage 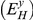 and the surface-area-specific somatic maintenance rate 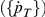. Recorded seawater temperatures in which juveniles were reared, declined from 28 to 20°C due to the approaching winter. Additional data on weight-length relationships in the early juvenile phase [29], together with information on freshly spawned egg size, secured estimates of the weight-energy coupler 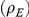 and shape factors (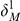 and 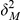).

Development of PBT broodstock cultivated at research station on Amami Island, southern Japan, provided data on growth in the adult stage [46]. Amami Island is characterized by relatively high seawater temperatures that range between 20 and 28°C. Similar range of seawater temperatures in all life stages ensured the comparability of growth data from different sources. Furthermore, comparable data allowed us to estimate the parameters that affect the entire lifecycle of PBT (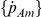, 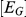, 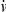, 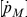 and 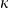) and whose values could not be fixed from an incomplete dataset. We also used recent measurements on PBT caught in the waters off Japan [47] to compare the growth in captivity and in the wild.

Batch fecundity was determined on sexually mature individuals caught by the Taiwanese small-scale longline fleet near the known spawning grounds at south-western part of North Pacific [44]. These data, together with information on age at maturity, provided a reference for assessing the parameters related to maturation and reproduction; the maturity maintenance rate coefficient 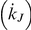, maturity at puberty 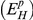 and average number of spawned batches during the reproductive season (*N*). We also used the number of spawned batches by the captive PBT individuals [46] to verify the estimated parameter values and discuss the results in the context of PBT reproductive season.

## Results

**Figure 2:**
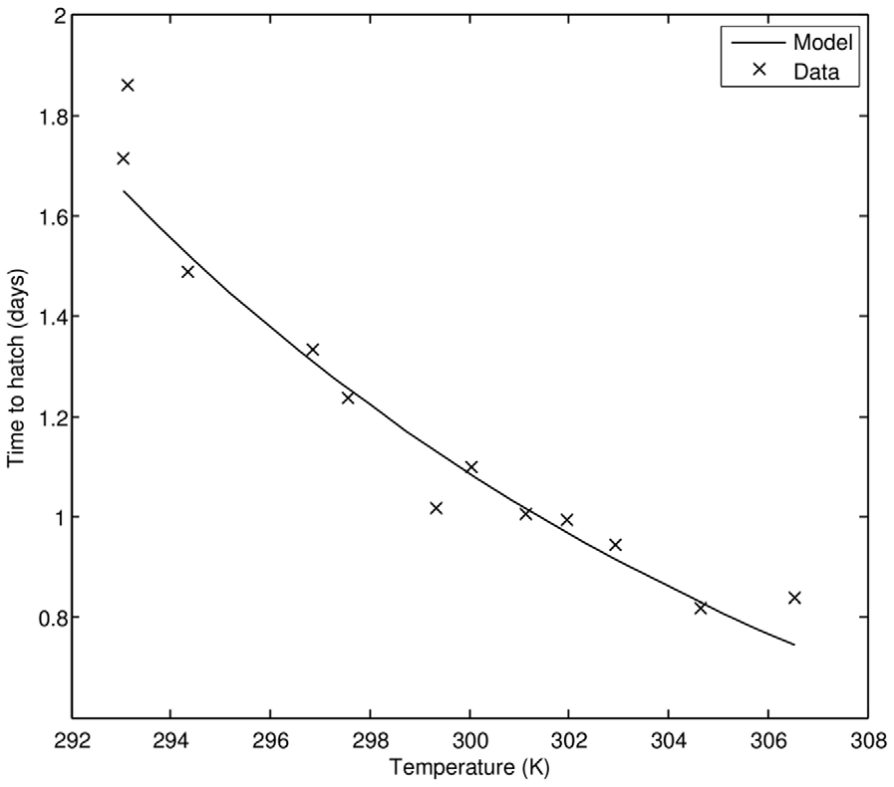
Time to hatching as a function of seawater temperature; comparison of model results to observations (data source: [**28**]).

Fully parameterized model reproduces many characteristics of the existing dataset on PBT. Comparison to univariate data in Table 7, for example, indicates satisfactory correspondence between the model and observations. Aspects of development in the embryonic stage (egg diameter and length at hatching) are particularly well captured. Additional evidence that the model provides a good description of embryonic development can be found by examining the hatching time vs. seawater temperature in Figure 2. As the seawater temperature increases from 293 K (around 20°C) to 306 K (33°C), the time it takes a freshly spawned egg to hatch decreases by more than 50%.

**Table 7:** Comparison of model results to univariate data.

Data	Model prediction	Literature value	Data source
Egg diameter	0.972 mm	0.926–1.015 mm	[29]
Length at hatching	3.07 mm TL	3.08 mm TL	[4]
Age at maturity	3.2 y	>3 y	[66]
Maximum physical length	327 cm FL	≈300 cm FL	[67]

**Figure 3:**
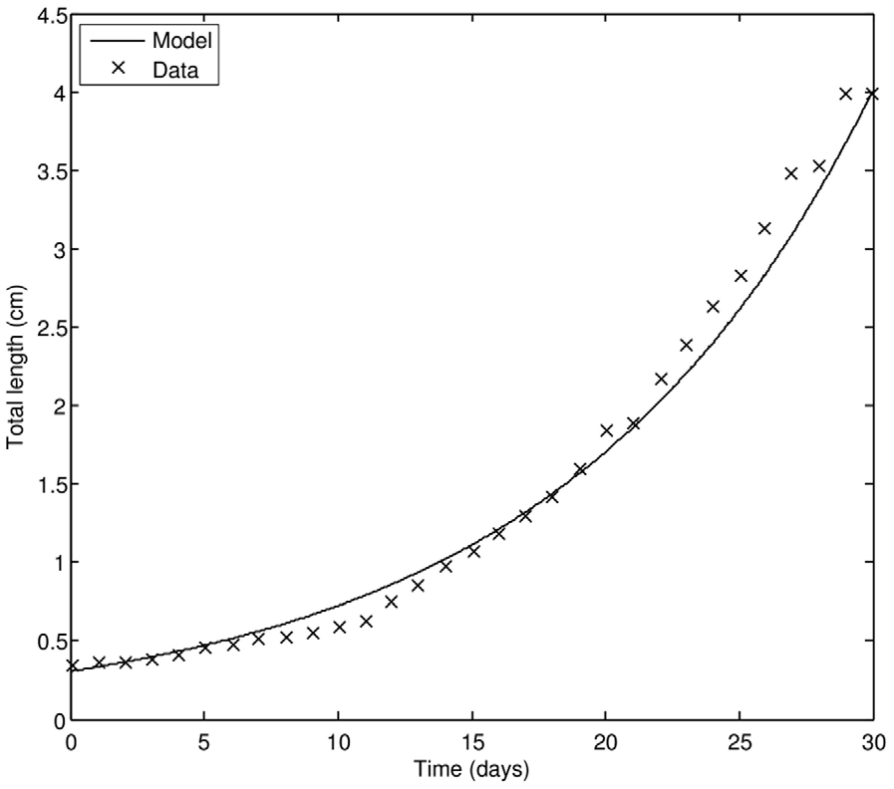
Growth of PBT in the larval stage; comparison of model results to observations (data source: [**4**]).

The model successfully captures the acceleration of growth rate in the larval stage, especially after 15–20 DAH (Figure 3). By the time larvae turn into young juveniles, the length of fish increases by a factor of 10 (from 4 mm to nearly 4 cm). We estimate that in controlled hatchery conditions the larval stage ends at approximately 36 DAH, at which point assimilation and utilization are as effective as in an adult individual.

**Figure 4:**
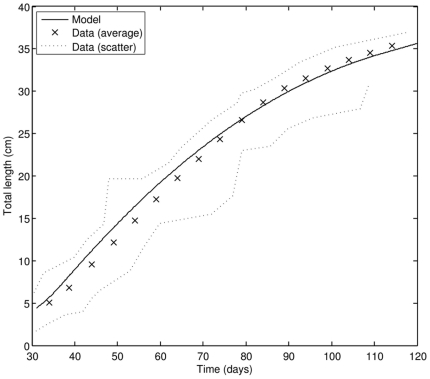
Growth of PBT in the early juvenile phase; comparison of model results to observations (data source: [**29**]).

Young PBT juveniles maintain very high growth rate of 0.45 cm d^−1^ up to 85–90 DAH (Figure 4). Toward the end of the early juvenile phase growth rate gradually drops. Our estimates show that in controlled hatchery conditions, the early juvenile phase ends at approximately 113 DAH. From this point on, internal heat production efficiency is maximal.

**Figure 5:**
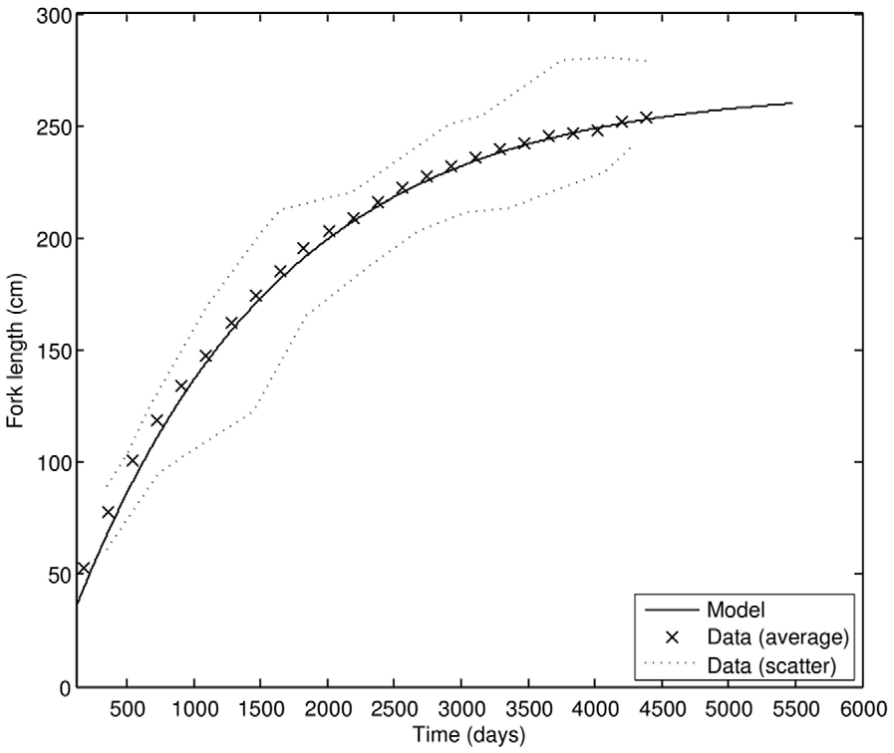
Growth of PBT in the adult stage; comparison of model results to observations (data source: [**46**]).

In the juvenile and adult stages, growth of cultivated PBT follows the von Bertalanffy curve with growth rates that are generally much lower than growth rates during the early juvenile phase. As the bluefin tuna approaches its ultimate size (which depends on food availability), the growth rate slowly decreases (Figure 5). From parameter values in Table 5, the von Bertalanffy growth rate in the adult stage is 0.249 y^−1^, while the expected ultimate size of an average PBT in cultivation is projected to be 267 cm FL. These values are close to the ones quoted in literature [46], confirming the consistency of estimated parameter values. The maximum possible size of PBT at food level 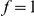, as predicted by the model, is 327 cm FL.

**Figure 6:**
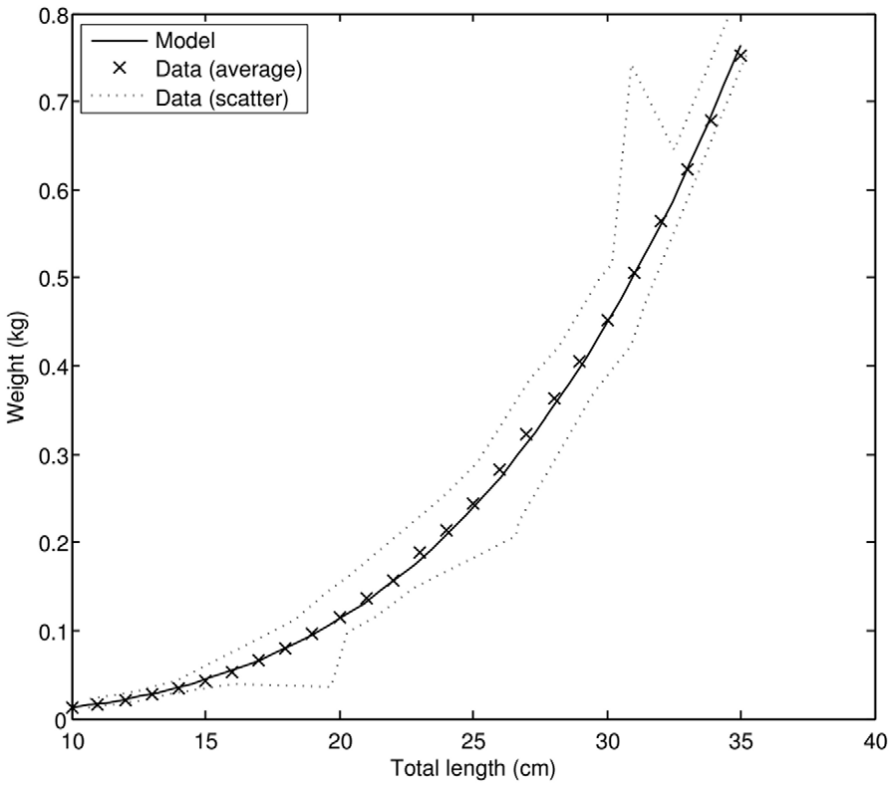
The weight-length relationship in the early juvenile phase; comparison of model results to observations (data source: [**29**]).

**Figure 7:**
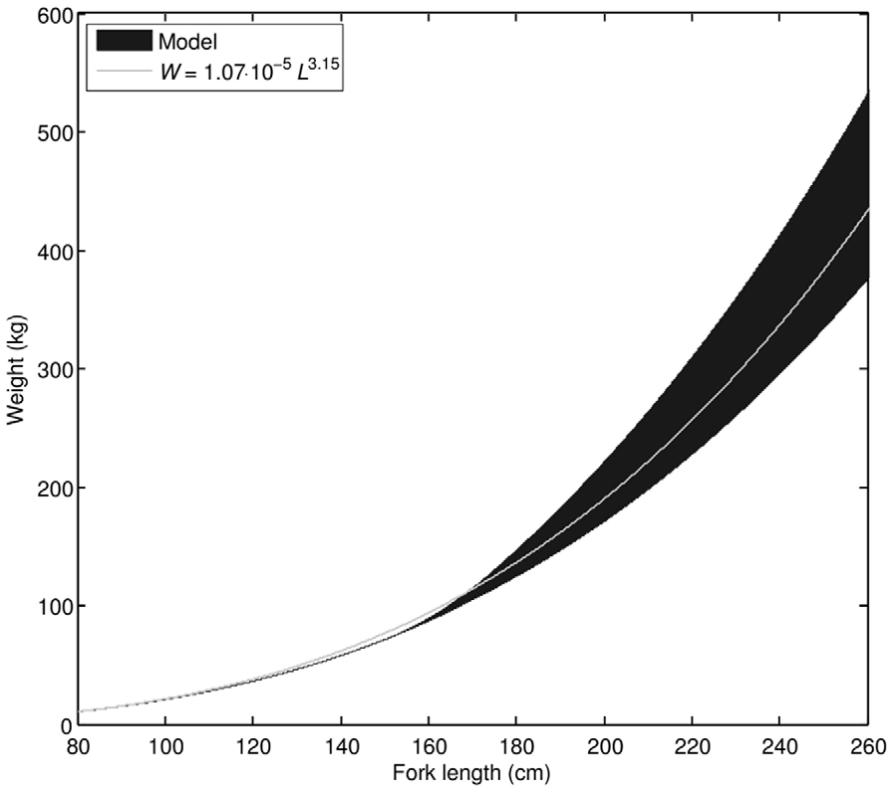
The weight-length relationship in the juvenile and adult stages; comparison of model results to the weight-length relationship available in literature [**46**]. Shaded area indicates that, even at constant food level, weight of an adult individual at a given length will vary greatly depending on the reproductive activity of the fish.

For a non-reproducing, strictly isomorphic organism at a constant food level, relation (**5**) shows that weight should scale with the cube of physical length. Data on weight-length relationship, however, indicate that in the early juvenile phase the scaling exponent between weight and physical length is considerably larger than 3 (our estimate is 3.32). The model successfully accounts for this difference (Figure 6) by assuming that PBT (i) rapidly change their body shape in the early life stages, especially prior to metamorphosis, and then (ii) asymptotically approach the final shape of an adult individual. In the adult stage, weight contribution of the reproductive buffer plays more and more significant role as the fish grows larger (Figure 7), suggesting that giant PBT (>180 cm FL) may experience a considerable weight loss during the reproductive season, when energy stored in the reproductive buffer is converted into eggs and released into the environment.

**Figure 8:**
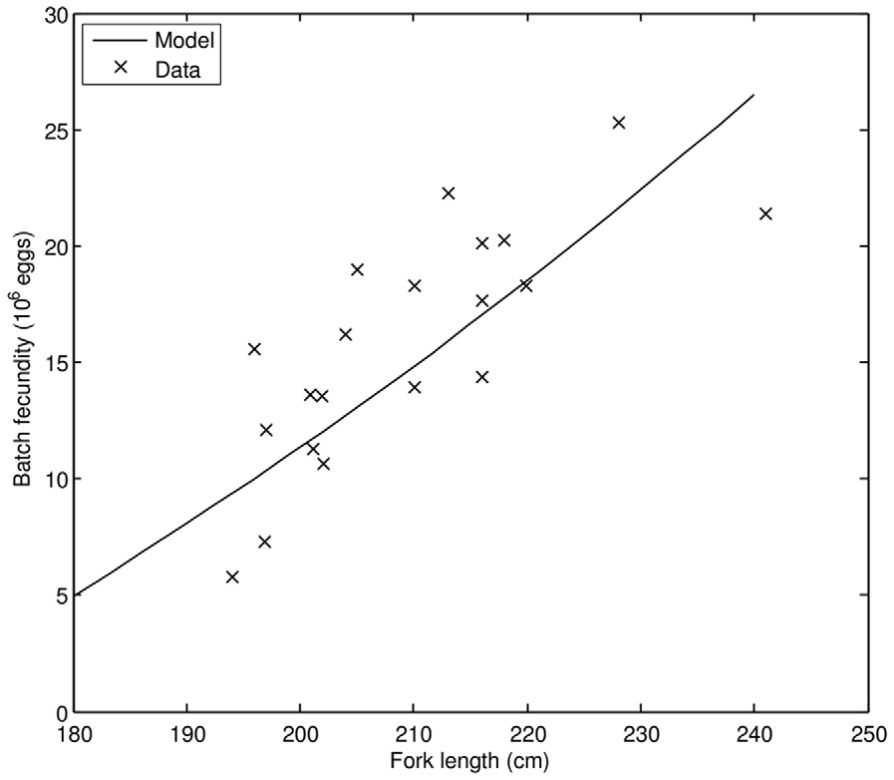
Batch fecundity as a function of fork length; comparison of model results to observations (data source: [**44**]).

In addition, we compared data on batch fecundity vs. fork length to model predictions (Figure 8). Again, the correspondence is good. The model showed that 3.2 years old PBT, corresponding to a cultivated fish of 150 cm FL, may achieve sexual maturity, but with relatively low batch fecundity. According to our estimates, giant PBT of 180 cm FL produces 5.0 million eggs that weigh approximately 2.4 kg per batch. As the fish grows to 240 cm FL, batch fecundity increases to 26.5 million eggs, weighing around 13 kg. We also estimated that the average number of batches per reproductive season, at food availability characteristic for cultivation, is 9. It was reported [46] that captive PBT had produced somewhere between 1 and 19 batches per reproductive season, suggesting that estimated average number of batches per reproductive season is consistent with observations.

**Figure 9:**
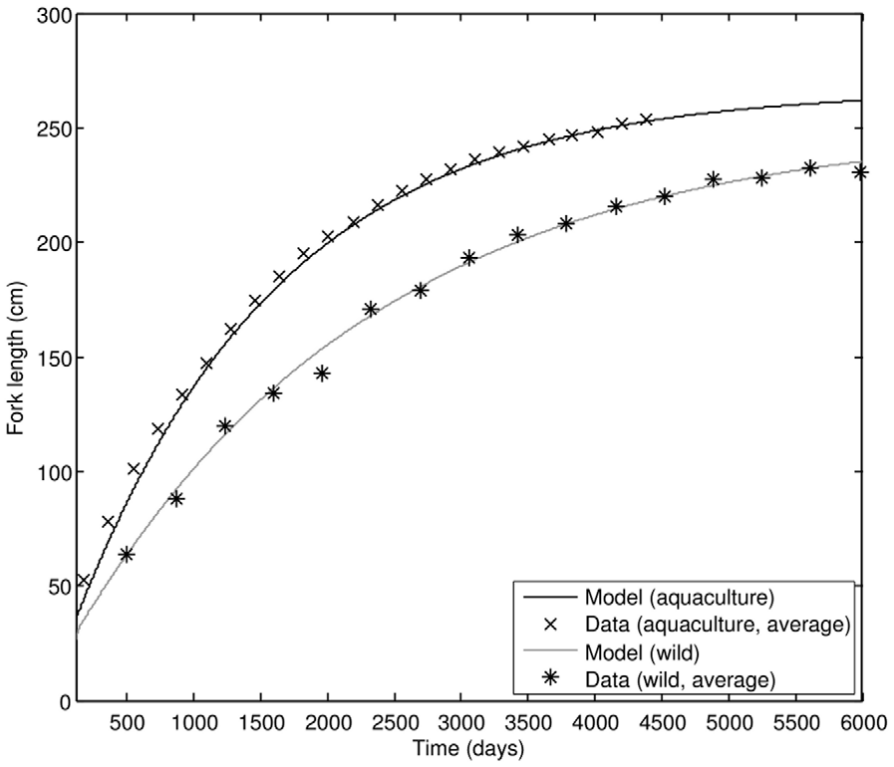
Growth comparison of cultivated and wild PBT (data sources: [**46]**, [47****]).

We simulated growth curves for cultivated and wild PBT by varying food availability and body temperature (Figure 9). Contrary to our expectations, temperature – not food availability – seems to be the primary cause for higher growth rates of cultivated PBT. Body temperature difference of around 6.5°C is sufficient to explain the observed difference in growth rates. Food availability, on the other hand, strongly affects the ultimate size of fish. Since the estimated ultimate size of wild PBT is 252 cm FL, comparison to cultivated fish (267 cm FL) indicates that daily consumption in the wild is only marginally smaller than in the aquaculture. Using equation (**1**), we estimated that cultivated fish experience food density equivalent to 12.3 times the half-saturation constant (i.e. 92.5% of satiation level). Wild fish, however, regularly experience food density equivalent to 9.5 times the half-saturation constant (90.5% of the satiation level). An interesting consequence of lower average body temperature and lower food availability is that the wild PBT reach sexual maturity later in life than cultivated PBT (152 cm FL, 5.3 years in the wild and 150 cm FL, 3.2 years in aquaculture).

**Figure 10:**
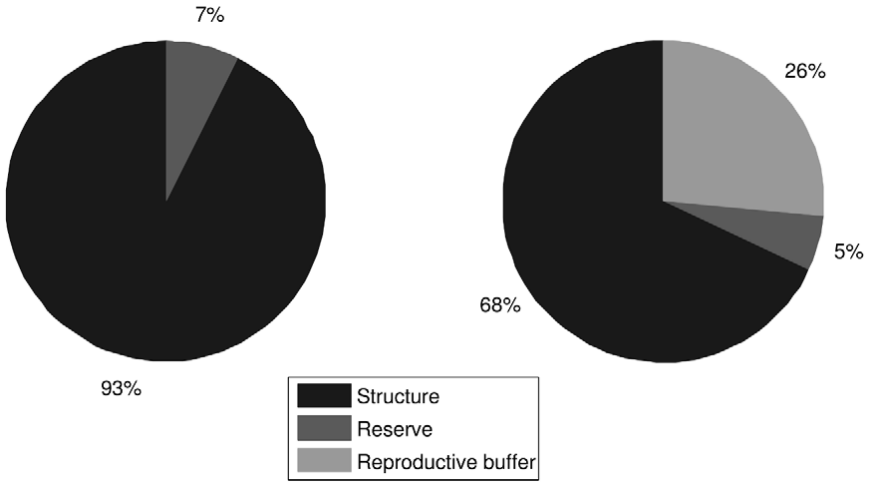
Relative contributions of structure, reserve and reproductive buffer to body weight of 220 cm long PBT prior to the beginning of the reproductive season (left panel; body weight 309 kg; condition index 2.9) and after the reproductive season (right panel; body weight 228 kg; condition index 2.1).

In Figure 10, we show how reproductive season affects the relative contributions of structure, reserve and reproductive buffer to body weight of a giant PBT of 220 cm FL. The most notable attribute is a large accumulation of energy for reproduction throughout the year. Energy accumulated for reproduction, represented in the model by the reproductive buffer, accounts for slightly more than 26% of body weight, or 82 kg out of 309 kg just prior to the reproductive season. If idealized handling of the reproductive buffer is assumed, where the release of energy accumulated for reproduction occurs instantaneously, 82 kg can be interpreted as the potential (i.e. an upper limit) for weight loss due to spawning. The second notable attribute of PBT is relatively low contribution of energy in reserve to body weight. At best, reserve accounts for slightly more than 7% of body weight, or 17 kg out of 228 kg right after the reproductive season.

**Figure 11:**
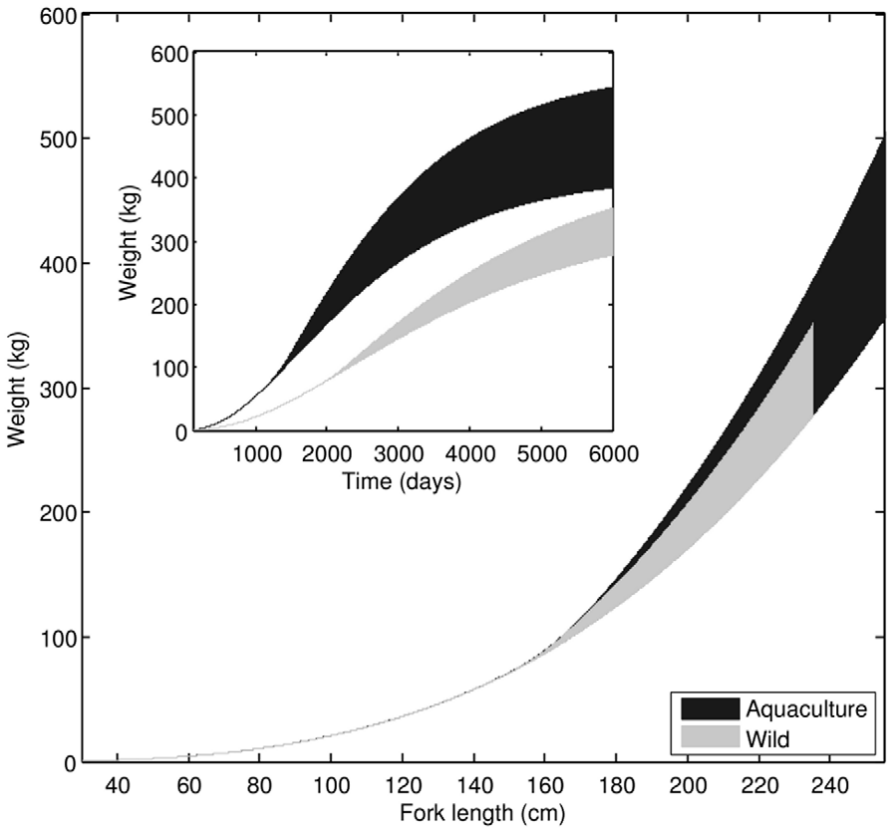
Comparison of weight-length relationships between cultivated and wild PBT as predicted by the model. Overlap of shaded areas indicate that for practical purposes these relationships are indistinguishable. Weight as a function of time, however, is quite different for cultivated and wild PBT (inlet).

Relatively low contribution of reserve to body weight has an interesting consequence; weight-length relationships of cultivated and wild PBT do not differ significantly (Figure 11). Overlap of possible weight ranges at food levels characteristic for aquaculture and the wild is consistent with observations that cultivated and wild PBT have “fairly similar” weight-length relationships [46]. If body weight is observed as a function of time, then the difference between cultivated and wild PBT is striking (Figure 11).

**Figure 12:**
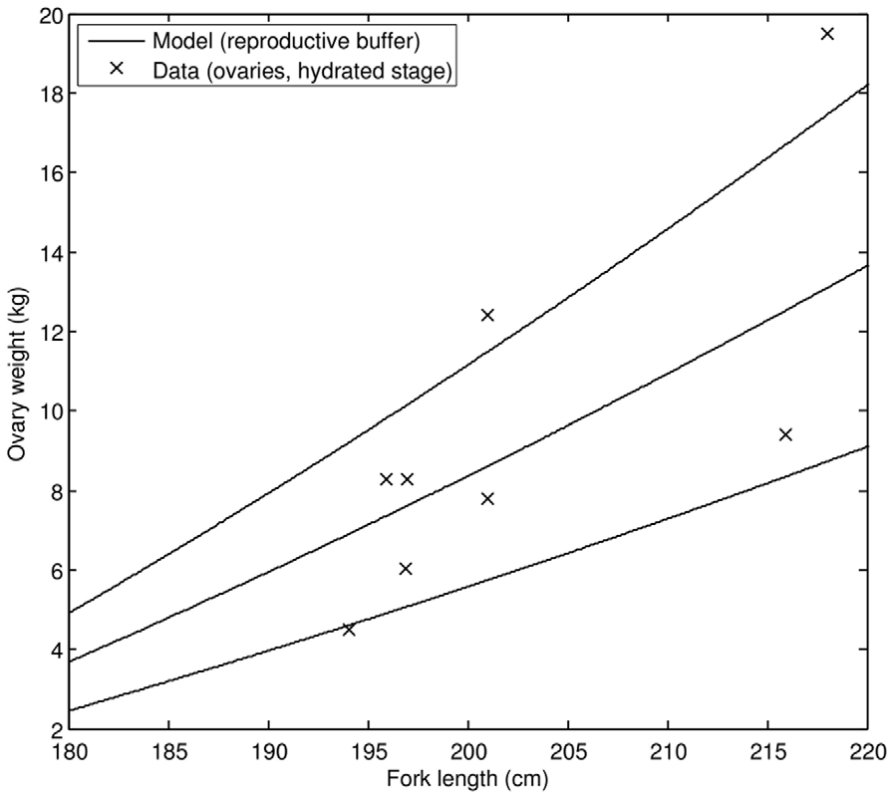
Comparison of predicted and observed ovary weight. Predictions were calculated from simulated reproductive buffer weight (see text). The weight ratio of non-hydrated to hydrated oocytes was set to 0 (the lowest line), 50% (middle line) and 100% (the uppermost line). Only measurements of ovaries with hydrated stage oocytes were selected for this comparison (data source: [44]).

We also compared predicted and measured ovary weights during the reproductive season (Figure 12). Predicted values were calculated from reproductive buffer weight assuming that the total weight of the buffer is equally divided among the average annual number of spawned batches (*N*). We used only data on ovaries with hydrated stage oocytes for comparison. Since such ovaries also contain a much larger number of oocytes in other stages of development (i.e. non-hydrated oocytes), predictions represent an estimate of the lower limit for observations. The comparison made here suggests that the weight ratio of non-hydrated to hydrated oocytes in ovaries of PBT can be as high as 100%, but the most likely weight ratio is around 50%. Similar weight ratios are implied by existing data when ovaries containing only non-hydrated oocytes are compared to ovaries with hydrated oocytes [44].

**Figure 13:**
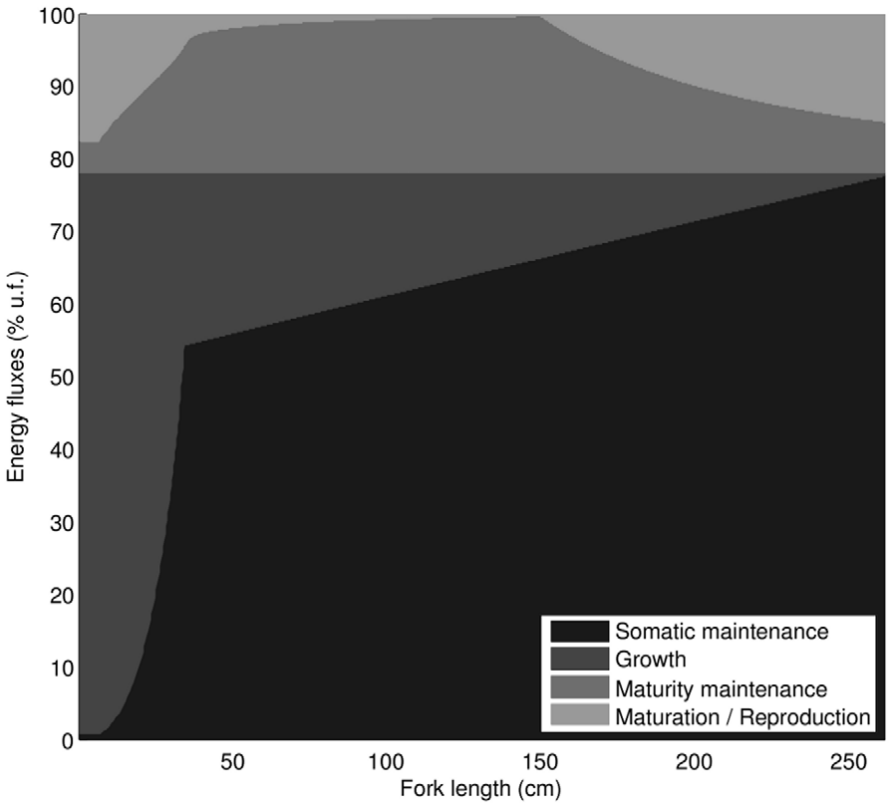
Energy fluxes relative to utilization flux as a function of fork length.

Allocation of energy during the lifetime of an individual PBT is represented by somatic maintenance, growth, maturity maintenance, maturation and reproduction fluxes relative to utilization flux (Figure 13). In the larval stage and early juvenile phase PBT expends most of utilized energy on growth and maturation, fueling the explosive increase in size and striking morphological and physiological changes. As the internal heating efficiency approaches maximum, maintenance costs start to dominate the overall energy budget, resulting in gradual deceleration of growth rate to significantly lower levels. For late juvenile and adult fish, the somatic maintenance increases with body size, leaving less and less energy for growth and causing the asymptotic advance toward the ultimate size which depends on food availability. Maturity maintenance costs become constant when fish enter the adult stage, allowing the investment of energy into reproduction to increase with size. The effect of κ-rule is visible by the constant proportion of somatic maintenance and growth fluxes in the utilization flux.

## Discussion

### The role of energy in reserve

Taking into account relatively small contribution of reserve to overall body weight of PBT, we investigated the growth potential based on energy in reserve. The ratio of volume-specific cost of structure 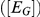 to maximum reserve energy density 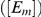 shows that increasing the structural volume by 1% requires about 13% of maximum energy that can be stored in the reserve. Obviously, the ability to grow solely on energy in reserve is quite limited.

If PBT cannot significantly grow using the reserve, can it at least survive prolonged starvation? To answer this, we need to relate somatic maintenance rate coefficients, 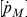 and 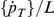, to maximum reserve energy density 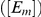. The new quantity is size-dependent and indicates the number of days a starving fish could survive if all possible energy in reserve is directed to somatic maintenance. For a fish of 40, 100 and 250 cm FL, maximum starvation times are 2.8, 6.6 and 13 days, respectively. Obtained values are extremely low, but consistent with observations. Starvation experiment on skipjack (*Katsuwonus pelamis*), kawakawa (*Euthynnus affnis*) and yellowfin tuna (*Thunnus albacares*) [48] indicated that within the first week of fasting a number of fish had shown signs of imminent death. Only one yellowfin tuna in the experiment survived over 30 days. Interestingly enough fish from all three species decreased the swimming speed almost to the minimum needed for maintaining neutral buoyancy, presumably as a measure of energy conservation. This fact suggests that tunas are capable of certain behavioral adjustments which may extend the survivability in starvation beyond our original estimates. More importantly, the ratio of final to initial energy content of the fish was non-linear in time, indicating severe starvation. Organisms with larger energy reserve would show linear response, because mobilized energy would have been utilized strictly for somatic maintenance purposes [16].

Perhaps the most obvious implication of limited ability to survive starvation is the necessity to act as a voracious predator. PBT simply needs to replenish the reserve at all times. This kind of reasoning is in line with previously stated result that wild PBT experience food availability equivalent to 9.5 times the half-saturation constant (that is, 90.5% of satiation level). Field studies performed on large wild Atlantic bluefin tuna (*Thunnus thynnus*, hereafter ABT) confirm that our claims are reasonable. ABT in the continental shelf waters off North Carolina shore consume primarily Atlantic menhaden (*Brevoortia tyrannus*) with daily ration reaching 2% of body weight per day [49]. For comparison, estimates show that the feeding rate of large ABT under fattening conditions in Mediterranean is 1.56% of body weight per day [50]. Conversion of these feeding rates into food level (*f*) and vice-versa depends on energetic content of feed and assimilation overhead following ingestion. Unfortunately, we could not determine the energetic content (or proximate composition) of Atlantic menhaden, but we were able to estimate that feed used for fattening of ABT has, on average, 5.28·10^6^ J kg^−1^ [51]. Applying this value to a giant PBT of 220 cm FL (weighing between 228 kg and 309 kg) at food level experienced in cultivation 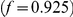, the model predicted feeding rate is between 1.24% and 1.65% of body weight per day in accordance with the observation under fattening conditions.

The assimilation overhead following ingestion is critically related to food quality. To understand why, let us assume that a compound A can be metabolized only in the presence of another compound B. Let us further assume that the compound A is in excess relative to the compound B due to a change in the available food. This kind of situation, in reality, does not lead to indefinite accumulation of compound A. If metabolic pathways allow, the compound A may be transformed into another form, which can then be stored or oxidized for energy. Fish, for example, have a low capacity for storing excess amino acids obtained from dietary protein, which results in the preferential removal of the α-amino group by fish hepatocytes and production of ammonia [52]. Ammonia represents a nitrogenous waste and an important contribution to the assimilation overhead. In aquaculture, therefore, diets are being carefully formulated to avoid losses and environmental issues associated with excess protein [53]. In relation to growth curves of fish, an increase in assimilation overhead leaves less assimilated energy in reserve for all metabolic processes, which strongly affects the ultimate body size. Since cultivated and wild PBT grow to a very similar ultimate size, there is no sufficient evidence to support the notion of lower food quality in the wild in comparison to cultivation.

Limited ability to survive starvation may also explain some properties of puzzling reproductive behavior of PBT. Unlike tropical tunas that exhibit protracted spawning seasons [34], [54], PBT and ABT reside in temperate waters and reproduce in designated spawning grounds for about one to two months a year [3], [44]. Concentrated reproductive efforts allow bluefin tunas to spend time feeding in richer, but colder, high latitude waters and turn back to spawning grounds when the conditions for larval development are optimal [55]. Therefore, the energy allocated to reproduction throughout the year is stored in the reproductive buffer and released only during a short time window of suitable environmental conditions. Accumulated energy for reproduction, as shown before, can amount to significant fraction of body weight in giant PBT. In view of limited ability to survive starvation, it is reasonable to expect that adult bluefin tunas can use energy from the reproductive buffer for survival if food availability is too low. In other words, bluefin tunas may have a mechanism that allows them to deplete the energy allocated to reproduction if survival is threatened. Confirmation that this mechanism is realistic comes from field studies showing the resorption of eggs (atresia) even as adult ABT individuals enter the spawning grounds in the Mediterranean Sea [56]. Furthermore, the same mechanism may be responsible for observations that adult ABT sometimes skip moving toward the spawning grounds during the reproductive season [3], [57], [58]. The existence of reproductive buffer, alongside possible behavioral adjustments, means that our initial estimates of starvation survivability are low-end values and that PBT are likely to survive somewhat longer periods without food.

### Physiological basis for growth rate acceleration during the larval stage

Research showing that PBT larvae rapidly develop their digestive system after hatching suggests that energy assimilation at constant food level improves as well. Many aspects of PBT larval development, including the digestive system, were summarized in literature [59]. Based on the state of notochord tip flexion, the larval stage is usually divided into three phases: pre-flexion, flexion, and post-flexion phase. PBT larvae in the pre-flexion phase have a primitive, larval type digestive system that allows feeding on miniature rotifers (0.1–0.5 mm lorica length). Gastric glands and pyloric caeca first appear in the flexion phase and noticeably increase in number toward the metamorphosis. Specific activity of trypsine-like and pepsine-like digestive enzymes also jumps markedly from the flexion phase, indicating a significant improvement of food assimilation thereafter.

Changes in the digestive system are paralleled by anatomical changes that suggest improvement of food ingestion after the flexion phase; preanal length, head length, head height, snout length, upper jaw length and eye diameter grow faster than the body length [29]. As a result, PBT larvae shortly after hatching develop into the typical scombrid larvae, characterized by disproportionally big head, large mouth and eyes, as well as an adult-type digestive system. The choice of suitable food also expands rapidly, first with *Artemia* nauplii (≥0.5 mm), then other fish larvae (e.g. striped knifejaw, ≥1.7 mm) and by the time of metamorphosis chopped fish meat [4], [29].

Apart from energy assimilation, two line of physiological evidence suggest that PBT larvae simultaneously improve energy utilization, even at constant food level. These evidence are the growth hormone activity and RNA to DNA (RNA∶DNA) ratio. Because an organism should not grow faster or slower than the rate at which needed energy and required metabolites can be mobilized from reserve, growth is carefully regulated by hormonal control [16]. Therefore, an increase in growth hormone activity should result in increased energy utilization. According to physiological studies, this is indeed the case in PBT, which has a very high %GH – defined as the ratio of growth hormone immunoreactive cells volume to pituitary volume – in comparison to other previously examined fish species [59]. More importantly, %GH increases from the post-flexion phase of PBT larval development, thus matching the occurrence of described digestive system and anatomical changes, and supporting the idea that efficiencies of energy assimilation and utilization improve at the same time.

In the context of RNA∶DNA ratio, if it is used as an indicator of protein synthesis, then the fact that RNA∶DNA in PBT increases steeply from 20 DAH into the early juvenile phase [59] suggests an increase in energy utilization. In line with previously brought up hormonal control of growth, higher mobilization rate from energy reserve would require faster incorporation of mobilized energy and elementary compounds into the new structure, which is a step where protein synthesis plays a crucial role. However, RNA∶DNA ratio may also be viewed as an indication of larval nutritional condition, because RNA can be used as a proxy for the amount of reserve, DNA as a proxy for the amount of structure and the ratio of reserve to structure increases with food availability [16]. Indeed, given the range of particles that PBT larvae can consume and considering differences in nutritional value of various larval feeds, it is easy to conceive that feeding level is not constant despite the fact that for artificially reared larvae laboratory conditions guarantee abundant food at all times. If, for example, larvae are limited by the size of particles they can consume, equation (**1**) may be modified so that effective food concentration depends on body size. This approach would certainly give enough flexibility to reproduce the observed near-exponential growth in the larval stage, but could not affect the egg development in any way. Given the high energy conductance of PBT, the model would predict that hatching occurs within few hours from spawning. On the contrary, our approach that assimilation and utilization are less effective until the end of the larval stage than in the adult stage leads to exact prediction of hatching time. This is the single most important piece of evidence in favor of equation (A1; Table 4).

### High Feed Conversion Ratio in bluefin tuna cultivation

Feed Conversion Ratio is an important quantity, widely used as a measure of how efficiently the fish convert feed mass into the fish biomass. In general, FCR is defined as the ratio of dry weight of supplied feed to gain in wet weight of the fish. FCR for large ABT (average initial and final weight 219–255 kg) under fattening conditions in the Mediterranean Sea can be as high as 7.4 on a dry-weight basis [50]. For smaller fish (average initial and final weight 32–63 kg) recorded FCR is 4.6. Even though FCR for smaller fish is much lower than FCR for adults, it is still high in comparison to other fishes. For example, Atlantic salmon (*Salmo salar*), divided into two groups depending on the fish size and fed four different diets, had conversion ratios between 0.97–1.24 for larger and 0.88–0.97 for smaller fish on a dry-weight basis [60].

To understand high FCR values in bluefin tuna cultivation, we must first acknowledge that the contribution of reserve to overall body weight is quite small. In immature fish, the reproductive buffer is negligible, because there is no allocation of energy to reproduction. Although the role of the reproductive buffer may be of some interest when considering FCR values of adult fish, the fraction of body weight attributable to structure remains dominant. In this context, the fact that the state of reserve, not structure, reacts directly to food availability means that any short term manipulation of food level would have little effect on FCR. Significant weight increases can be attained only through the growth of structure. Thus, it is necessary to examine the allocation of assimilated energy to growth. Our simulations suggest that, on average, growth flux amounts to less than 14% of assimilation flux during the period in which weight increases from 30 to 60 kg (113 to 142 cm FL). Hence, PBT allocate a very small proportion of assimilated energy to growth. We hypothesize that low allocation is the main reason why bluefin tuna cultivation records such high FCR in comparison to other fishes. Furthermore, the possibility of lowering FCR is quite limited. Some improvements could perhaps be achieved by the development of high-energy artificial feeds [61].

### Additional notes on reproductive biology of Pacific bluefin tuna

Due to inherent difficulties in simultaneous tracking of the position and the reproductive status of bluefin tunas, little is known about the amount of time a single individual spends inside the spawning grounds while being reproductively active. Observations made in captivity show that PBT can spawn over protracted time-span in one year (May–June, September–October), but spawning period and number of produced batches are quite irregular. In 2001, for example, individuals from a PBT broodstock reared on Amami Island, south Japan, produced between 1 and 19 batches [46].

To give an estimate of the time period a single fish spends inside the spawning grounds while being reproductively active, we use the model results and existing data on spawning period of bluefin tunas. According to our calculations PBT in favorable conditions can produce an average of 9 batches a year, where number of eggs per batch increases with fish size. Since variability of batch fecundity is quite high (e.g. Figure 8), situations in which fish spawn more than 9 batches a year with fewer than expected eggs (and vice versa) could easily occur. If we take 9 to be a representative average number of batches in one year and assume the spawning period of 2–4.5 d [44], an individual PBT will be reproductively active between 18 and 40.5 d y^−1^. This result affirms the notion that PBT focus their reproductive effort to a short time window of suitable environmental conditions. Therefore, understanding what environmental factors trigger what changes in hormonal control of reproductive behavior might be the key to successful production of larvae in captivity.

In the case of PBT individuals of older age, which are very close to their ultimate size, it is sometimes believed that metabolic expenditures preclude the fish from effective spawning [58]. This phenomenon can be viewed in the context of predicted energy allocation (Figure 13) and discussed mechanism of energy depletion from the reproductive buffer in critical times. The somatic maintenance costs, which in large fish already drain a big portion of energy from reserve, may temporarily increase after the periods of high activity (such as spawning) when the fish incurs a significant oxygen debt. Recouping the oxygen debt in the largest individuals can last for days and require energy from the reproductive buffer in the process, ultimately leading to a conservative spawning behavior at older age.

From a bioenergetic point of view, we do not expect the reproductive biology of bluefin tuna to differ significantly between the sexes. For example, a histological study on testicular development in migrant and spawning ABT [62] found a significant negative correlation between the gonad and fat tissue indices, suggesting the mobilization of reserve from the reproductive buffer for gametogenesis as the reproductive season approaches. In the context of testes maturation, five adult PBT males, ranging in size between 133.8 cm TL and 144.4 cm TL and estimated to be between 2.6 and 2.8 years old, were observed in captivity at the Ohshima Experimental Station of the Fisheries Laboratory of Kinki University, Kushimoto, Japan [63]. This observation may indicate that males enter the adult stage somewhat earlier than females, although the same study reports a 151.4 cm TL male individual with testes still in development. At present, it is not clear to which extent are these young individuals reproductively active.

### Model improvements and future development

At the current stage of model development we only estimate the energy allocated to the reproductive buffer and assume that this energy is released instantaneously once a year. Such handling of the reproductive buffer is idealized, because it neglects the fact that PBT are reproductively active between 18 and 40.5 d y^−1^ during which the feeding does not cease. As a consequence, the weight loss implied by Figure 10 is overestimated. The predicted value represents the potential weight loss, which can be viewed as an upper limit for the real weight loss observed in form of seasonal changes in the condition index [44], [47]. In addition, the lack of more detailed rules to handle the energy stored in the reproductive buffer precludes us from predicting the spawning period. If the appropriate handling rules for the reproductive buffer were devised, the model could be used to estimate the impact of environmental conditions on the spawning period and thus the duration of the entire spawning season [19], [20].

Predicting tuna population dynamics could help manage their stock. A DEB model could provide an opening to population dynamics [17], first by studying the equilibrium state. Using reproductive rates from a DEB-based model with mortality data and setting the average lifetime reproductive output to 1, it is possible to estimate the food density at which population reaches an equilibrium. Afterwards, various demographic properties can also be estimated. To move beyond the equilibrium state, it would be necessary to link a DEB-based model to a structured population dynamics model, for example by integrating DEB into matrix population models [64] or following developments of cohorts [65].

### Conclusion

The model presented herein estimates the metabolic rates of an individual PBT throughout its entire lifetime, thereby providing new insights into physiology and ecology of this invaluable species. In particular, we demonstrated that:

1. Difference in body temperature – not food level – can explain the differences in growth of wild and cultured PBT.
2. Average energy intake in the wild is adequate to support the yearly reproductive cycle. Moreover, if intake were uninterrupted, reproduction could occur throughout the year. Since the wild PBT concentrate their reproductive effort to a short time period, suitable environmental conditions seem to play an important role in triggering egg production.
3. Meeting huge maintenance demands of PBT requires regular and sizable energy intake to avoid the depletion of energy reserve. In this context, bluefin tunas could be described as an extreme case of a demand system [16]. Since PBT would experience serious difficulties in meeting maintenance requirements at low food levels, possible growth curves for this species are highly restricted, indicating a minimum level of metabolic flexibility. Behavioral flexibility, on the other hand, is expanded maximally by physiological specializations like regional endothermy, which permits bluefin tunas to explore productive, high latitude areas.
4. PBT are poorly equipped to deal with starvation; a characteristic that is likely to reflect both on feeding and reproductive behavior. Estimated food level indicates that PBT are efficient predators in the wild. However, should feeding conditions become unfavorable large PBT are quickly forced to rely on behavioral adaptation (e.g. slower swimming) and energy that is normally reserved for reproduction (e.g. atresia).
5. Acceleration of growth rate in the larval stage could be accounted for by hypothesizing an improvement in energy assimilation and utilization as a consequence of morphological and physiological transformations prior to metamorphosis. This hypothesis represents a novel attempt to understand the observed growth pattern of PBT in the larval stage.
6. Deceleration of growth rate by the end of the early juvenile phase can be explained by the onset of internal heat production. This hypothesis represents a novel attempt to understand the observed growth pattern of PBT in the early juvenile phase.

Our results emphasize the need for further research into the (i) effects of food-size limitation on growth of PBT in the larval stage, (ii) development of artificial feeds and feeding strategies that might reduce high FCR observed in aquaculture (iii) environmental factors that control the reproductive behavior of PBT, and (iv) relations between an individual PBT and population-scale demographic properties.

## Supporting Information

Appendix A **List of symbols.**
(DOC)

Appendix B **Derivation of equations F2 and D2.**
(DOC)
