# Supplementary material for: A Full Lifecycle Bioenergetic Model for Bluefin Tuna
Source: PLoS One. 2011 Jul 11;6(7):e21903. doi: 10.1371/journal.pone.0021903 (PMC3133599; doi:10.1371/journal.pone.0021903)
Supplement: Appendix A — List of symbols. (DOC) [file pone.0021903.s001.doc]

# Appendix A: List of symbols

*d_S_* – Density of structure

*E* – Amount of energy in reserve tissue

*E*_0_ – Initial energy reserve of an egg

$\left[ E_{G} \right]$ – Volume specific cost of structure

*E_H_* – Level of maturity

$E_{H}^{2}$ – Half-saturation maturity, i.e. the level of maturity at which the shape factor is an arithmetic mean of $\delta_{M}^{1}$ and $\delta_{M}^{2}$

$E_{H}^{b}$ – Maturity at birth

$E_{H}^{j}$ – Maturity at the end of the larval stage

$E_{H}^{p}$ – Maturity at puberty

$E_{H}^{y}$ – Maturity at the end of the early juvenile stage

$\left[ E_{m} \right]$ – Maximum reserve energy density,

*E_R_* – State of the reproductive buffer, i.e. energy accumulated for reproduction between two reproductive seasons

*E_Rm_* – State of the reproductive buffer just prior to the reproductive season

*f* – Holling type II functional response

*K* – Condition index

$\dot{k}_{J}$ – Maturity maintenance rate coefficient

*L* – Structural volumetric length; when cubed gives the structural volume ($V=L^{3}$)

*L_b_* – Structural volumetric length at birth

*L_j_* – Structural volumetric length at metamorphosis

$M_{1}$ – Shape correction function

$M_{2}$ – Efficiency of internal heat production

*N* – Average number of spawned batches during the reproductive season

$\dot{p}_{A}$ – Assimilation flux

$\dot{p}_{C}$ – Utilization (mobilization, catabolic) flux

$\dot{p}_{G}$ – Growth flux

$\dot{p}_{J}$ – Maturity maintenance flux

$\dot{p}_{M}$ – Volume related somatic maintenance flux

$\dot{p}_{R}$ – Maturity or reproduction flux

$\dot{p}_{S}$ – Somatic maintenance flux

$\dot{p}_{T}$ – Surface-area related somatic maintenance flux

– Maximum surface-area-specific assimilation rate

– Volume-specific somatic maintenance rate

– Surface-area-specific somatic maintenance rate

*T* – Body temperature

$T_{0}$ – Reference temperature; set to $T_{0}=298.65\text{ K}$ roughly reflecting the conditions in which PBT were reared

$T_{A}$ – Arrhenius temperature

*T_a_* – Ambient (environmental, seawater) temperature

*V* – Volume of structural tissue

$\dot{v}$ – Energy conductance

*W* – Body weight

*W_egg_* – Weight of an egg

*X* – Food density

*X_K_* – Half-saturation constant, i.e. food density at which $f=0.5$

$\delta_{M}$ – Shape factor

$\delta_{M}^{1}$ – Shape factor in the larval stage

$\delta_{M}^{2}$ – Shape factor in the adult stage

$\kappa$ – Fraction of mobilized reserve allocated to soma

$\rho_{E}$ – Weight-energy coupler
