# Supplementary material for: A Full Lifecycle Bioenergetic Model for Bluefin Tuna
Source: PLoS One. 2011 Jul 11;6(7):e21903. doi: 10.1371/journal.pone.0021903 (PMC3133599; doi:10.1371/journal.pone.0021903)
Supplement: Appendix B — Derivation of equations F2 and D2. (DOC) [file pone.0021903.s002.doc]

# Appendix B: Derivation of equations Error: Reference source not found and Error: Reference source not found

Rigorous derivation of equation (Error: Reference source not found; Error: Reference source not found) follows from the basic assumptions of DEB theory [Error: Reference source not found, Error: Reference source not found]. A somewhat simplified approach is also presented in literature [Error: Reference source not found]. From Error: Reference source not found, the mass balance for the reserve density, $\left[ E \right]=E/V$, can be written

, (B1)

In DEB theory, reserve density determines the chemical composition of organisms; a property directly related to the homeostasis assumption. Therefore, the homeostasis assumption greatly restrict the reserve density dynamics in the sense that, at constant food level $f=f^{'}$, the chemical composition of organisms does not change. After introducing the structural length $V=L^{3}$, and noting that , the homeostasis assumption requires that

, (B2)

because at $f=f^{'}$ the reserve density does not depend on size, i.e. , where and are yet undetermined functions. Let us first determine the function $\dot{G}$ by noting that the reserve density $\left[ E^{'} \right]$ depends on food level $f^{'}$, while the utilization of reserve is assumed to be only a function of state variables, i.e. (intuitively, the reserve “filters” high frequency changes occurring in the environment and secures the stability of internal conditions). This is possible only if $\dot{G}=0$. Comparing equations (B1) and (B2) now yields

. (B3)

A simplified approach [Error: Reference source not found] determines the function $\dot{H}$ by directly assuming the first order regulatory process for the reserve density dynamics, i.e. , where $\dot{v}$ is the model parameter. More formal approaches [Error: Reference source not found, Error: Reference source not found] consider the metabolic stoichiometry, which states that reserve is transformed into structure (*V*) and metabolites, *M*_1_ to *M_z_*, according to the (stylized) chemical reaction

, (B4)

with *c_G_*, *a*_1_ to *a_z_* being the stoichiometric coefficients in C-moles. If reserve is divided into $\lambda_{1}E$ and $\lambda_{2}E$, where , then

, (B5)

must be valid, because otherwise the chemical composition of reserve will change – a violation of the homeostasis assumption. This also implies . From equation (B3), for a fully grown adult, we then have , which means that the regulatory process for the reserve density is indeed a first order process, i.e. . Inserting the last expression into equation (B3) results in a relationship equivalent to (Error: Reference source not found; Error: Reference source not found). To include the shape correction, a substitute $\dot{v}\to M_{1}\dot{v}$ is necessary. Subsequent application of the κ-rule produces the rightmost expression in (Error: Reference source not found; Error: Reference source not found).

Equation (Error: Reference source not found; Error: Reference source not found) is a mathematical statement of assumption that a part of mobilized reserve is invested into the growth of structure (Error: Reference source not found). Since the chemical composition of reserve and structure differs, to produce a unit of structure *V* costs $\left[ E_{G} \right]$ units of energy from reserve. This can be written

. (B6)

Introducing the structural length, $V=L^{3}$, into the above equation results in

, (B7)

which is equivalent to equation (Error: Reference source not found; Error: Reference source not found).
